# A phase II study of sequential treatment with anthracycline and taxane followed by eribulin in patients with HER2-negative, locally advanced breast cancer (JBCRG-17)

**DOI:** 10.1007/s10549-021-06396-0

**Published:** 2021-09-23

**Authors:** Ippei Fukada, Yoshinori Ito, Naoto Kondo, Shoichiro Ohtani, Masaya Hattori, Eriko Tokunaga, Nobuki Matsunami, Kohjiro Mashino, Taijiro Kosaka, Masahiko Tanabe, Daisuke Yotsumoto, Kosho Yamanouchi, Masataka Sawaki, Masahiro Kashiwaba, Hidetaka Kawabata, Katsumasa Kuroi, Satoshi Morita, Shinji Ohno, Masakazu Toi, Norikazu Masuda

**Affiliations:** 1 Department of Breast Medical Oncology, Breast Oncology Center, The Cancer Institute Hospital of the Japanese Foundation for Cancer Research, 3-8-31, Ariake, Koto-ku, Tokyo, 135-8550 Japan; 2 Department of Breast Surgery, Nagoya City University Hospital, Nagoya, Aichi Japan; 3 Department of Breast Surgery, Hiroshima City Hiroshima Citizens Hospital, Hiroshima, Japan; 4 Department of Breast Oncology, Aichi Cancer Center, Nagoya, Aichi Japan; 5 Department of Breast Oncology, National Kyusyu Cancer Center, Fukuoka, Japan; 6 Department of Breast Surgery, Osaka Rosai Hospital, Osaka, Japan; 7 Department of Breast Surgery, Shuto General Hospital, Yamaguchi, Japan; 8 Department of Surgery, Oita Prefectural Hospital, Oita, Japan; 9 Department of Breast Surgery, Juntendo University Nerima Hospital, Tokyo, Japan; 10 Department of Breast Surgery, Hito Medical Center, Ehime, Japan; 11 Department of Breast Oncology, Juntendo University Hospital, Tokyo, Japan; 12 Department of Breast and Endocrine Surgery, The University of Tokyo Hospital, Tokyo, Japan; 13 Department of Breast Surgery, Social Medical Corporation Hakuaikai, Sagara Hospital, Kagoshima, Japan; 14 Department of Surgery, Nagasaki University Graduate School of Biomedical Sciences, Nagasaki, Japan; 15 Department of Breast Oncology, Breastopia Miyazaki Hospital, Miyazaki, Japan; 16 Adachi Breast Clinic, Kyoto, Japan; 17 Department of Breast and Endocrine Surgery, Toranomon Hospital, Tokyo, Japan; 18 Department of Breast Surgery, Tokyo Metropolitan Cancer and Infectious Diseases Center, Komagome Hospital, Tokyo, Japan; 19 Tokyo Metropolitan Health and Hospitals Corporation Ebara Hospital, Tokyo, Japan; 20 Department of Biomedical Statistics and Bioinformatics, Kyoto University Graduate School of Medicine, Kyoto, Japan; 21 Breast Oncology Center, The Cancer Institute Hospital of the Japanese Foundation for Cancer Research, Tokyo, Japan; 22 Breast Surgery, Kyoto University Graduate School of Medicine, Kyoto, Japan; 23 Department of Surgery, Breast Oncology, NHO Osaka National Hospital, Osaka, Japan

**Keywords:** Eribulin, Neoadjuvant chemotherapy, Sequential therapy, HER2-negative, Locally advanced breast cancer

## Abstract

**Purpose:**

The sequence of taxanes (T) followed by anthracyclines (A) as neoadjuvant chemotherapy has been the standard of care for almost 20 years for locally advanced breast cancer (LABC). Sequential administration of eribulin (E) following A/T could provide a greater response rate for women with LABC.

**Methods:**

In this single-arm, multicenter, Phase II prospective study, the patients received 4 cycles of the FEC regimen and 4 cycles of taxane. After the A/T-regimen, 4 cycles of E were administered followed by surgical resection. The primary endpoint was the clinical response rate. Eligible patients were women aged 20 years or older, with histologically confirmed invasive breast cancer, clinical Stage IIIA (T2–3 and N2 only), Stage IIIB, and Stage IIIC, HER2-negative.

**Results:**

A preplanned interim analysis aimed to validate the trial assumptions was conducted after treatment of 20 patients and demonstrated that clinical progressive disease rates in the E phase were significantly higher (30%) than assumed. Therefore, the Independent Data Monitoring Committee recommended stopping the study. Finally, 53 patients were enrolled, and 26 patients received the A/T/E-regimen. The overall observed clinical response rate (RR) was 73% (19/26); RRs were 77% (20/26) in the AT phase and 23% (6/26) in the E phase. Thirty percent (8/26) of patients had PD in the E phase, 6 of whom had achieved cCR/PR in the AT phase. Reported grade ≥ 3 AEs related to E were neutropenia (42%), white blood cell count decrease (27%), febrile neutropenia (7.6%), weight gain (3.8%), and weight loss (3.8%).

**Conclusion:**

Sequential administration of eribulin after the A/T-regimen provided no additional effect for LABC patients. Future research should continue to focus on identifying specific molecular biomarkers that can improve response rates.

## Introduction

Neoadjuvant chemotherapy (NAC) for breast cancer has been used mainly for locally advanced cancer aiming at down-staging. The NSABP B-18 and B-27 trials showed similar overall survival (OS) and disease-free survival (DFS) rates between pre-operative and postoperative chemotherapy regimens [1, 2]. Preoperative docetaxel administration increased the pathological complete response (pCR) rate by 12% [2, 3]. In patients in whom pCR was obtained after NAC, both the OS and DFS rates were good [1]. Currently, the pCR rate is used as an important parameter to assess the therapeutic effects of preoperative chemotherapy. As prognostic factors for pCR to preoperative chemotherapy, hormone receptor (HR)-negative and histological grade 3 have been reported [4, 5]. However, no improvement in DFS or OS was seen, despite an improvement in the pCR rate. In NSABP B-27, AC and AC-DTX improved the pCR rate by 13%, but there was no improvement in the prognosis [6]. According to reviews of preoperative chemotherapy in Japan, the pCR rate for FEC administration after PTX therapy was 27.7%, and the response rate was 85.1% [7]. After FEC therapy, that for DTX administration was 25%, and the response rate was 75% [8]. Currently, as standard perioperative chemotherapy for breast cancer, sequential therapy with anthracycline and taxane preparations (A–T therapy) is performed; a more potent treatment has not been established. Recently, research on biomarkers to select patients for whom preoperative chemotherapy may be effective has been promoted. However, neither clinicopathological nor molecular biological markers to predict the tumor-reducing effects or long-term prognosis in preoperative chemotherapy for breast cancer have been sufficiently examined. Especially in those with a larger tumor diameter or more advanced cancer, it is difficult to achieve the disappearance of cancer cells using these drugs. Therefore, recently, as a new therapeutic strategy, A–T therapy simultaneously or sequentially combined with a new drug was proposed to improve clinical response and radical surgery rates. In the Gepar Trio Trial, the subjects were divided into two groups (responders and non-responders) after preoperative TAC therapy (two courses), and the effects of additional treatment were examined. The responders were divided into two groups receiving TAC × 6 (conventional arm) and TAC × 8 (response-guided arm), respectively. The sonographic response rates were 75.2% and 74.1%, respectively. The physical response rates were 86.3% and 83.5%, respectively [9]. On the other hand, the non-responders were divided into two groups receiving TAC × 6 (conventional arm) and NX (response-guided arm), respectively. The sonographic response rates were 50.5% and 51.2%, respectively (difference: 0.7 percentage points, *p* = 0.004). Those receiving NX showed significantly better results [10]. Recently, a report from the Gepar Trio Trial on long-term prognosis (SABCS2011) showed that NX (response-guided arm) prolonged DFS and OS more markedly than the conventional arm. In both the responders and the non-responders, the response-guided arm also showed better results. Furthermore, it improved the prognosis, with a DFS difference of approximately 10 percentage points after 5 years, whereas the difference in the response rate was 0.7 percentage points. In the Gepar Trio Trial, the establishment and concept of treatment differed from those in this study. However, these results suggest that, when the effects of standard treatment are not marked, another new regimen may improve the results of treatment. As described above, various efforts have been made to achieve better response in NAC for locally advanced breast cancer, Seung et al. reported the 5-year survival rate for Stage III breast cancer is 70.2%, indicating a poor prognosis [11]. Therefore, in the case of inoperable locally advanced breast cancer, the standard chemotherapy regimens including anthracycline and taxane are currently not satisfactory.

Recently, the efficacy of a new microtubule inhibitor, eribulin, was evaluated in patients with breast cancer, and it was confirmed that eribulin significantly prolonged survival in AT-treated patients with advanced/recurrent breast cancer in comparison with conventional drugs selected by attending physicians. A phase II study of eribulin in Japan was conducted in subjects in whom the median number of previous chemotherapeutic regimens involving A and T preparations was 3 (1–5), of which 2 (0–3) were following progression/recurrence**.** The response rate was 21.3% [12]. And Inoue et al. reported eribulin monotherapy was effective and well tolerated in heavily pretreated patients with metastatic breast cancer who had well-defined taxane resistance [13]. Therefore, the results of standard chemotherapy with A and T preparations are not satisfactory in patients with locally advanced breast cancer (LABC) in whom surgery is impossible, and the tumor size may be more markedly reduced by sequentially combining eribulin with this therapy and thus prolonging survival.

The purpose of this study was to examine the clinical response rate of patients with advanced, local, HER2-negative breast cancer to sequential therapy with anthracycline, taxane, and eribulin. In addition, the histological effects, safety, and clinical usefulness were investigated. Clinical usefulness was evaluated using the proportion of patients undergoing radical surgery, the proportion of those undergoing breast-preserving surgery, relapse-free survival, and OS as parameters. In a sub-study, the relationship between the antitumor effects of eribulin and biological profiles of cancer tissue was examined using molecular biological and biochemical procedures.

## Methods

### Patients

Patients with LABC of stage IIIA (only T2–3/N2 are eligible), stage IIIB, and stage IIIC were recruited. All patients participating in this study had been histologically confirmed to have primary invasive adenocarcinoma of the breast with the tumor negative for HER-2 expression as determined by a local hospital laboratory (score of 0 or 1 on an immunohistochemical test [range 0–3, with a score of 0 or 1 indicating HER2-negative breast cancer, a score of 2 a marginal result, and a score of 3 HER2-positive breast cancer; in the case of a marginal result, HER2 status was examined by means of fluorescence in situ hybridization to establish a positive or negative result] or a negative result on fluorescence in situ hybridization). Both protein and genetic status were estimated based on the guidelines for HER2 testing in breast cancer, as edited by American Society of Clinical Oncology/College of American Pathologists [14]. Other key eligibility criteria were age of 20–70 years, Eastern Cooperative Oncology Group (ECOG) performance status score of 0 or 1 (on a 5-point scale, with higher numbers indicating greater disability), previously untreated (irradiation, chemotherapy, endocrine therapy, immunotherapy, or molecular targeted therapy) for LABC, and patients who could provide written, informed consent.

Key exclusion criteria were the presence of breast cancer in bilateral breasts, other malignant conditions or synchronic multiple cancers, other concurrent serious disease that may interfere with planned treatment, including severe pulmonary conditions/illness, uncontrolled infections, uncontrolled diabetes, or uncontrolled hypertension, pregnant or lactating, hypersensitivity to any of the study medications, and chronic immunosuppressive therapies, including systemic corticosteroids.

### Trial design and oversight

**Fig. 1:**
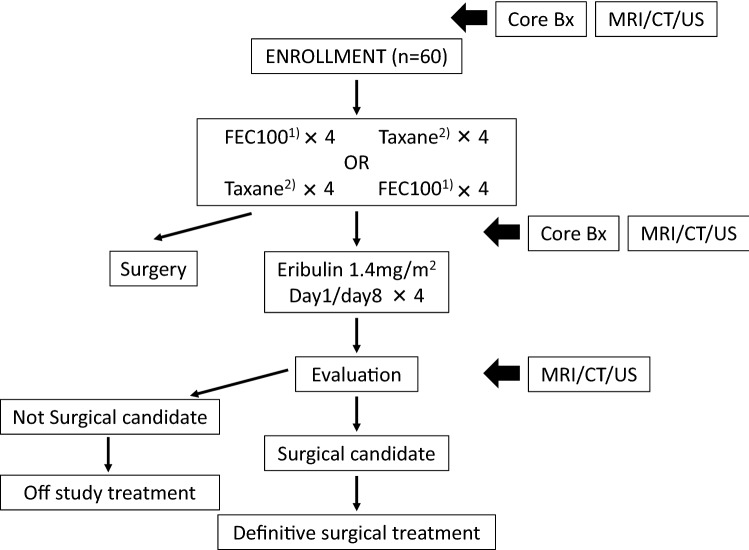
Study design. A single arm phase II study, preoperative sequential therapy with anthracycline and taxane followed by eribulin in patients with HER2-negative, locally advanced breast cancer. ^1)^FEC100; fluorouracil 500 mg/m^2^, epirubicin 100 mg/m^2^, and cyclophosphamide 500 mg/m^2^. ^2)^Taxane therapy, docetaxel 75 mg/m^2^, weekly paclitaxel 80 mg/m^2^, or nab-paclitaxel 260 mg/m^2^ was used

As preoperative therapy, eribulin therapy was performed after anthracycline/taxane (AT) therapy: sequential AT therapy with FEC100 (4 cycles) and taxane (4 cycles) (Fig. 1). For anthracycline therapy, FEC100 was basically selected, but FEC75 or AC (60/600) was also possible, considering safety and age. For taxane therapy, DOC75 (4 cycles), weekly PTX 80 (4 cycles), or nab-PTX 260 (4 cycles) was used. The order of FEC100 and taxane administration was selected by the attending physicians. For eribulin therapy, 1 cycle consisted of 21 days. Eribulin at 1.4 mg/m^2^ was administered on Days 1 and 8, and discontinued until Day 21. After the completion of the 4th cycle of eribulin therapy, antitumor effects were examined by surgery or tissue biopsy. When surgery was impossible, or when there was no evidence of adverse reactions affecting the continuation of administration in the absence of disease progression, the administration of eribulin was continued.

In Japan, eribulin is approved for the treatment of inoperable locally advanced or metastatic breast cancer following the use of anthracycline and taxane regimen. This study was planned for high-risk patients with stage IIIA (only T2–3/N2 are eligible), stage IIIB, and stage IIIC locally advanced breast cancer who was judged to be unresectable, and since eribulin was administered after the use of anthracycline and taxane regimen, it was determined that the study could be conducted under the Japanese universal insurance.

### End points

The primary end point of the trial was the clinical response rate to sequential therapy with anthracycline, taxane, and eribulin in women with HER-2 negative LABC. Secondary end points included the pCR rate of the A/T/E regimen, the safety of the A/T/E regimen, OS and DFS of patients treated with the A/T/E regimen, and the response rate to the A/T/E regimen.

### Imaging modalities for determining treatment effect

Imaging evaluation should be performed after sequential chemotherapy with anthracycline and taxane, and at the end of eribulin therapy. Imaging evaluation was performed using palpation, ultrasonography, CT or MRI, and the overall efficacy was determined based on RECIST ver1.1.

### Definition of pathological complete response (pCR)

In this study, pCR was defined as no invasive residual in breast or nodes; noninvasive breast residuals allowed (ypT0/is ypN0).

### Statistical analysis

The efficacy end point was the clinical response rate. According to a review on preoperative chemotherapy in Japan, Toi et al. reported the pCR was achieved in 25% of patients treated with DTX after FEC therapy. The response rate was 75% [15]. A phase II study of eribulin in Japan was conducted in subjects in whom the median number of previous chemotherapeutic regimens involving the A /T regimen was 3 (1–5), with a median of 2 (0–3) after progression or recurrence, and the response rate was 21.3%. In the study by Toi et al. mentioned above, the majority of the patients had T1 or T2 tumors (79.8%), and 99.5% of patients were N0 or N1. However, our study included high-risk patients with stage IIIA (only T2–3/N2 are eligible), stage IIIB, and stage IIIC. Based on these points of view, it was anticipated that neoadjuvant treatment with eribulin following an A/T-regimen would produce a clinical response rate of at least 70%, and a sample size of 47 evaluable patients was required (one-sided test of hypothesis; power of 80%; *α* = 0.05). Accrual of 60 patients was planned to allow for a 20% unevaluable rate. A preplanned interim analysis that aimed to validate the trial assumptions was conducted after treatment of 20 patients, and it demonstrated that the clinical progressive disease (cPD) rate to the A/T/E regimen was significantly higher (30%) than assumed.

## Results

### Patients’ characteristics

**Fig. 2:**
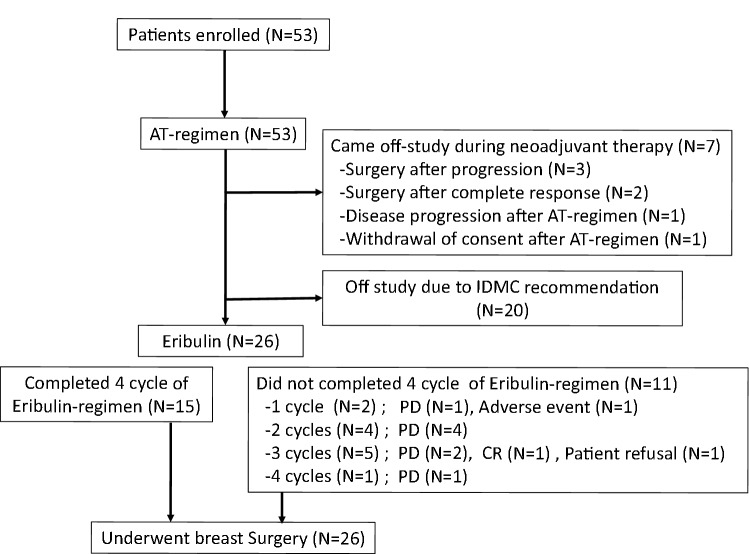
Study consort diagram. A total of 53 patients were enrolled, 7 patients were came off-study during neoadjuvant therapy and 20 patients were off study due to IDMC recommendation. Finally, 26 patients received the A/T/E-regimen

The CONSORT diagram of the study is shown in Fig. 2. A preplanned interim analysis that aimed to validate the trial assumptions was conducted after treatment of 20 patients, and it demonstrated that the cPD rate in the E phase was significantly higher (30%) than assumed. Therefore, the Independent Data Monitoring Committee recommended stopping the study. A total of 53 patients were enrolled, and 26 patients received the A/T/E-regimen. The baseline demographic and clinical characteristics of the patients (27 patients of AT phase only and 26 patients of AT-E phase) are shown in Table 1. For these 26 patients, the median age at diagnosis was 49 (32–67) years, 69% (18/26) of the patients were hormone receptor (HR)-positive, and 31% (8/26) of the patients were HR-negative. The clinical tumor stage was T2 in 10 (39%), T3 in 9 (35%), and T4 in 7 (27%). The clinical nodal status was N1 in 5 (19%), N2 in 7 (27%), and N3 in 14 (54%). Clinical staging was as follows: Stage IIIA 23% (6/26); Stage IIIB 23% (6/26); and Stage IIIC 54% (14/26).

**Table 1:** Patients’ characteristics (AT phase only and AT-E phase)

	AT phase only (27 patients, 28 target lesion)		AT-E phase (26 patients)	
	No.	%	No.	%
Age (years)				
50 >	11	40.7	15	57.7
50 and more	16	59.3	11	42.3
Menopausal status				
Premenopausal	12	44.4	15	57.7
Postmenopausal	15	55.6	11	42.3
PS				
0	26	96.3	25	96.2
1	1	3.7	1	3.8
Clinical tumor size				
T2	7	25.0	10	38.5
T3	8	28.6	9	34.6
T4	13	46.4	7	26.9
Clinical nodal stage				
N1	6	21.4	5	19.2
N2	6	21.4	7	26.9
N3	16	57.1	14	53.8
Clinical stage				
IIIA	4	14.3	6	23.1
IIIB	8	28.6	6	23.1
IIIC	16	57.1	14	53.8
ER/PR status				
ER+/PR+	13	46.6	13	50.0
ER+/PR−	5	17.9	5	19.2
Triple negative	10	35.7	8	30.8

### Clinical response

The clinical response rates (RRs) for each group are shown in Table 2. The overall clinical response was CR in 3 patients (11.5%), PR in 13 patients (50.0%), PD in 8 patients (30.8%), and SD in 2 patients (7.7%); the overall RR was 61.5% (16/26 patients).

**Table 2:** Clinical response

	Overall	AT phase	Eribulin phase
All subtype (*n* = 26)			
CR	3 (11.5%)	2 (7.7%)	3 (11.5%)
PR	13 (50.0%)	18 (69.2%)	3 (11.5%)
PD	8 (30.8%)	2 (7.7%)	8 (30.8%)
SD	2 (7.7%)	4 (15.4%)	10 (38.5%)
NE	0	0	2 (7.7%)
Luminal (*n* = 18)			
CR	3 (11.5%)	2 (7.7%)	2 (7.7%)
PR	8 (30.8%)	10 (38.5)	1 (3.8%)
PD	1 (3.8%)	2 (7.7%)	3 (11.5%)
SD	5 (19.2%)	3 (11.5%)	9 (34.6%)
NE	2 (7.7%)	1 (3.8%)	3 (11.5%)
Triple negative (*n* = 8)			
CR	0	1 (3.8%)	0
PR	6 (23.1)	5 (19.2%)	2 (7.7%)
PD	1 (3.8%)	0	3 (11.5%)
SD	0	2 (7.7%)	2 (7.7%)
NE	1 (3.8%)	0	1 (3.8%)

*CR* complete response, *PR* partial response, *PD* progressive disease, *SD* stable disease, *NE* not evaluable

In the AT phase, the RR was 76.9% (20/26), with CR in 2 patients (7.7%), PR in 18 patients (69.2%), PD in 2 patients (7.7%), and SD in 4 patients (15.47%). In the E phase, the RR was 23.1%, with CR in 3 patients (11.5%) and PR in 3 patients (11.5%); 30.8% (8/26) of patients had progressive disease, with SD in 10 patients, (38.5%) and N.E. in 2 patients (7.7%).

**Fig. 3:**
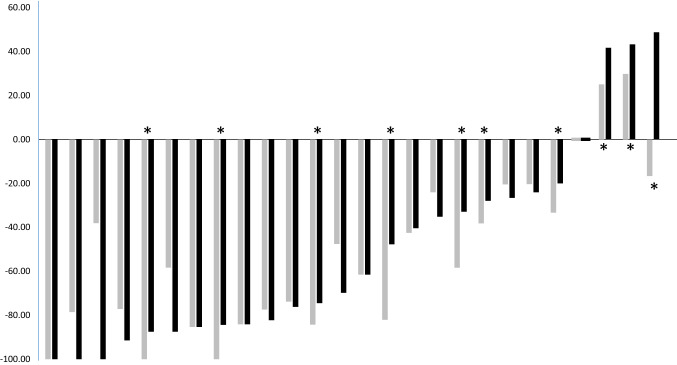
Waterfall plots of the percent change in tumor size. The Waterfall plots showing change in tumor size after the initial anthracycline and taxane regimen from baseline (gray bar) and after completion of additional eribulin treatment from baseline (black bar) by RECIST v1.1 criteria. Asterisks (*) indicate patients with disease progression during the periods of eribulin course

The Waterfall plots are shown in Fig. 3, showing change in tumor size after the initial anthracycline and taxane regimen from baseline (gray bar) and after completion of additional eribulin treatment from baseline (black bar) by RECIST v1.1 criteria.

### Pathological response

In 27 patients (28 lesions) who were treated with A–T alone, no pCR was obtained. Of the 26 patients who could be treated with A–T–E, pCR was observed in 4 of 25 patients except one patient who refused surgery, and the pCR rate was 16% (95% CI 5.8–35.35).

### Adverse events

All 26 patients who received the A/T/E-regimen were included in the safety analysis (Table 3). In the A/T phase, the common hematologic adverse events were leukopenia, anemia, and neutropenia. The common non-hematologic adverse events in the A/T phase were fatigue, occurring in 20 patients (76.9%), edema (73.1%), peripheral sensory neuropathy (69.2%), nausea (61.5%), constipation (57.7%), stomatitis (53.8%), and nail change (53.8%). Most adverse events were grade 1 or 2 in severity. In the A/T phase, neutropenia of grade 3 or 4 was noted in 34.6% of the patients, leukopenia of grade 3 or 4 in 38.5%, and anemia of grade 3 or 4 in 38.5%. Febrile neutropenia was noted in 2 patients (7.7%).

**Table 3:** Adverse events

Adverse event, *n* (%)	AT phase (*n* = 26)		Eribulin phase (*n* = 26)	
	All grades	Grade 3/4	All grades	Grade 3/4
Non-hematologic toxicities				
Fever	11 (42)	0	3 (12)	0
Fatigue	20 (77)	0	18 (69)	0
Nausea	16 (62)	0	8 (31)	0
Vomiting	4 (15)	0	0	0
Anorexia	6 (23)	0	4 (15)	0
Stomatitis	14 (54)	0	3 (12)	0
Diarrhea	11 (42)	0	3 (12)	0
Edema	19 (73)	0	17 (65)	0
Constipation	15 (57)	0	7 (27)	0
Arthralgia	10 (39)	0	5 (19)	0
Myalgia	10 (39)	0	6 (23)	0
Nail changes	14 (54)	0	10 (39)	0
Weight loss	1 (4)	0	5 (19)	1 (4)
Peripheral sensory neuropathy	18 (69)	0	14 (54)	0
Hematologic toxicities				
Neutropenia	9 (35)	17 (65)	10 (38)	11 (42)
Leukopenia	10 (38)	11 (42)	13 (50)	7 (27)
Anemia	10 (38)	0	9 (35)	0
Febrile neutropenia	0	2 (8)	0	2 (8)

In the E phase, the common hematologic adverse events were also leukopenia, anemia, and neutropenia. The common non-hematologic adverse events in the E phase were fatigue, occurring in 18 patients (69.2%), edema (65.4%), peripheral sensory neuropathy (53.8%), nail change (38.5%), nausea (30.8%), and constipation (26.9%). In the E phase, neutropenia of grade 3 or 4 was noted in 38.5% of the patients, leukopenia of grade 3 or 4 in 50.0%, and anemia of grade 3 or 4 in 34.6%.

## Discussion

The present results showed that there was no additional effect of sequential administration of eribulin following the anthracycline and taxane-regimen for patients with LABC. Although eribulin has clinical activity against metastatic breast cancer resistant to taxane, this study failed to demonstrate eribulin’s clinical activity when given immediately subsequent to taxanes and anthracycline in the neoadjuvant setting. In metastatic breast cancer, eribulin is able to produce an equivalent PFS as first or second-line treatment compared with taxane [16]. In contrast, administration of eribulin in the neoadjuvant setting showed less pathological response compared with taxane [17]. These paradoxical activities of eribulin in different settings could account for the negative result seen in the present study.

Possible causes of the difference in the results of pre-operative eribulin treatment for early breast cancer and those of eribulin treatment for metastatic breast cancer include the following factors. First, eribulin has different modes of action: one is a blockade of cell cycle progression by inhibition of microtubule growth, and the other is non-mitotic complex effects on tumor biology, including induction of vascular remodeling, suppression of cancer cell migration and invasion, and reversal of the epithelial-to-mesenchymal transition [18–24]. Short-term exposure to eribulin of a bulky mass with stage III would not be enough to improve the tumor microenvironment. In terms of immediate tumor reduction, eribulin may be inferior to taxane. Eligible patients with stage III LABC require the maximum best response to obtain long-term survival. Other approaches to LABC should be investigated. However, in the adjuvant setting after tumor resection, a residual small tumor burden may allow activation of vascular remodeling by eribulin. A phase II trial of postoperative eribulin in breast cancer patients who did not achieve pCR following standard neoadjuvant chemotherapy showed that it was feasible and well tolerated [25]. However, that study did not achieve its targeted 2-year DFS endpoints (improvements of 40%, 15%, and 25% in TNBC, ER+, and HER2+, respectively) in any of the three cohorts treated, and the 2-year DFS in each cohort was similar to the result in the control arms of several large studies in which patients not achieving pCR received no subsequent adjuvant treatment.

Second, there might be cross-tolerance between taxane and eribulin with immediate sequential exposure in a neoadjuvant setting for patients with LABC. In the EMBRACE Study [26], which examined patients who had undergone anthracycline- and taxane-based treatment for MBC, eribulin showed a 2.5-month extension of the median OS in patients who were taxane-refractory (median OS was 13.1 months for eribulin monotherapy and 10.6 months for TPC). However, Inoue et al. evaluated the clinical efficacy of eribulin in MBC patients who had well-defined taxane resistance, and they reported that the PD rate was 49.0% in a phase II, multicenter, single-arm, open-label clinical study [13].

Although the translational research was prepared in this study, only 7 of the patients who consented to the translational research were able to complete to A–T–E treatment and obtain specimens before and after treatment. Therefore, the translational research was discontinued because no useful conclusions could be obtained.

The present study had a limitation. This study was planned to include both luminal and triple negative breast cancer patients. Seung et al. reported the survival rates and median overall survival by breast cancer subtype. In their report, the 5-year survival rate for stage III triple negative breast cancer is 47.2%. Although the prognosis is 72.9% for the luminal type, it is not favorable [11]. In HER2-positive breast cancer, based on the results of the APHINITY trial, sequential administration of docetaxel + trastuzumab + pertuzumab and anthracyclines is commonly used in locally advanced breast cancer, and a therapeutic strategy of sequential administration of T-DM1 has been developed as an additional treatment for patients who have failed to achieve pCR with preoperative chemotherapy. Under these circumstances, there is less possibility of developing sequential therapy with eribulin for HER2-positive locally advanced breast cancer. As mentioned above, eribulin has shown efficacy in HER2-negative metastatic breast cancer that is resistant to AT therapy, and eribulin could be an effective treatment option for high-risk locally advanced breast cancer with a poor prognosis. Since the position of eribulin in the HER2-positive and HER2-negative groups is different, it is important to conduct a study focusing on the HER2-negative group.

This study was conducted as an exploratory pilot study in a high-risk, locally advanced HER2-negative group with a poor prognosis. Depending on the results of this study, we could expect to explore the possibility of leading to further clinical trials targeting patient groups who might be expected to benefit from sequential administration of eribulin.

In summary, based on these features of eribulin and the present results, we do not recommend further development of sequential administration of eribulin following an anthracycline and taxane-regimen in the neoadjuvant setting for patients with LABC. Future research should continue to focus on identifying specific molecular biomarkers, which can improve response rates.

## Conclusion

A preplanned interim analysis intended to validate the trial assumptions was conducted after treatment of 26 enrolled patients. The cPD rate after the eribulin-regimen was 30.8%, and the pCR rate was 16%. Toxicity was evaluated in 26 patients. The combination was safe, with mostly grade 1 and 2 toxicities. Because of the high cPD rate in response to the eribulin regimen, the study was terminated because of futility. Future research should continue to focus on identifying specific molecular biomarkers that can improve response rates.

## Data Availability

Not applicable.
